# Pancreatitis of ectopic pancreatic tissue: a rare cause of gastric outlet obstruction

**DOI:** 10.1093/gastro/gov037

**Published:** 2015-07-29

**Authors:** Saleh Elwir, Brooke Glessing, Khalid Amin, Eric Jensen, Shawn Mallery

**Affiliations:** 1 Department of Gastroenterology and Hepatology, University of Minnesota, Minneapolis, MN, USA; 2 Department of Pathology, University of Minnesota, Minneapolis, MN, USA; 3 Department of Surgical Oncology, University of Minnesota, Minneapolis, MN, USA

**Keywords:** gastric outlet obstruction, ectopic pancreas, pancreatitis

## Abstract

Inflammation in ectopic pancreatic tissue can clinically present with pain or obstructive symptoms, depending on the location of the ectopic tissue. We present a rare case of gastric outlet obstruction secondary to pancreatitis of ectopic pancreatic tissue in the pylorus.

## Introduction

Heterotopic pancreas is defined as pancreatic tissue that lacks anatomical or vascular communication with the normal body of the pancreas [1]. Most commonly it is an incidental finding of no clinical significance; however, it may come to notice when complicated by pathological changes such as inflammation, bleeding and malignant transformation [2]. We report a case of gastric outlet obstruction that resulted from pancreatitis of ectopic pancreatic tissue that was located in the pylorus.

## Case presentation

**Figure 1.**
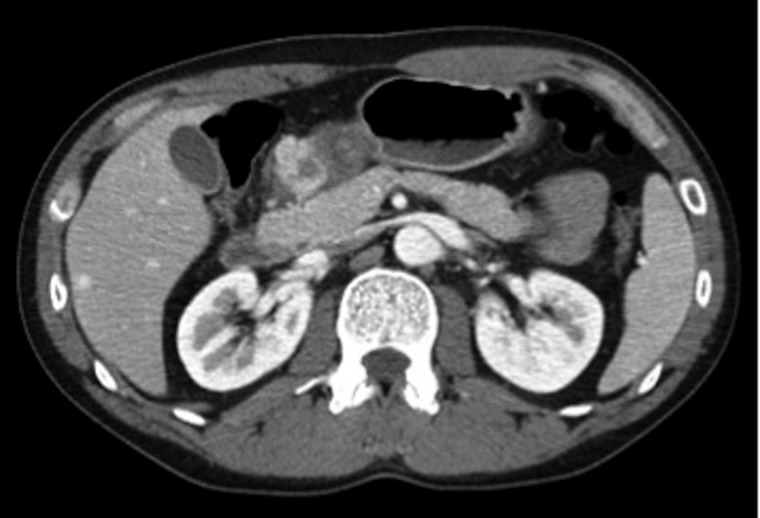
Thickening in pyloric wall, with an enhancing lesion in the proximal duodenum. Note sharply demarcated pancreatic borders with no stranding.

A thirty-five-year-old male patient presented to the hospital for evaluation of 3 months of nausea, vomiting and a 25 lb weight loss. The patient was initially seen for these symptoms at an another hospital, at which point his laboratory test results were unremarkable with the exception of an elevated lipase at 1900 U/L (normal range 23–300 U/L). The patient had no significant past medical history and he reported alcohol use of up to 18 cans of beer per week over the past 10 years. A computed tomography (CT) scan of the abdomen and pelvis was performed; it did not reveal any evidence of pancreatic or peripancreatic edema but showed wall thickening, edema and abnormal enhancement in the region of the pylorus and gastric antrum (Figure 1). The patient was managed conservatively and later discharged home.

**Figure 2.**
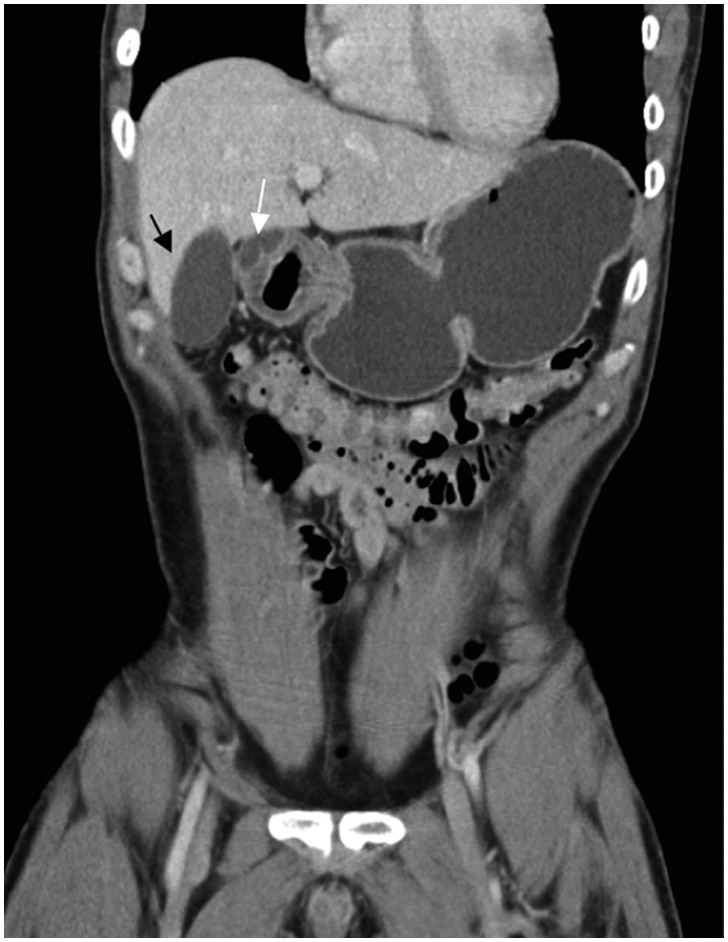
Thickening of the pyloric wall with an intramural fluid collection (white arrow), black arrow represents gall bladder.

The patient’s symptoms continued to progress and he presented to this hospital for further evaluation. His laboratory results on presentation showed a mildly elevated lipase (471 U/L) (normal range 20–250 U/L), with an unremarkable complete blood count, electrolytes and liver panel. A repeat CT scan of the abdomen and pelvis with intravenous contrast showed thickening and enhancement of the gastric pylorus and proximal first duodenal segment, with a well-defined fluid collection within the wall (Figure 2).

**Figure 3.**
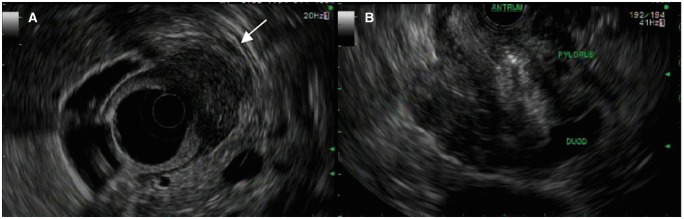
(A) Radial EUS scope showing hypoechoic lesion involving the submusosa and muscularis propria (white arrow). (B) Linear EUS scope showing asymmetric thickening of pyloric wall.

**Figure 4.**
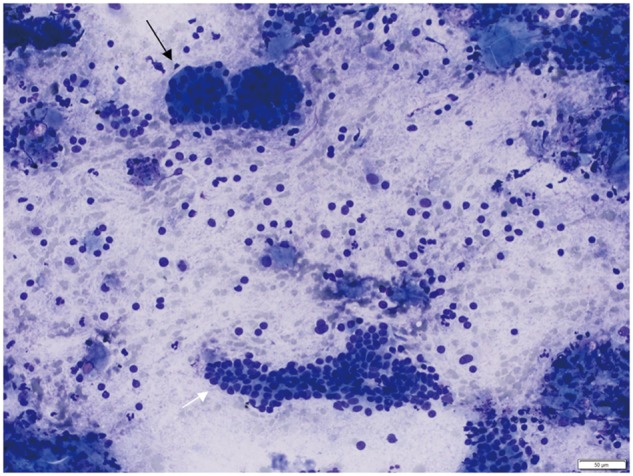
Acinar cells (black arrow) and ductal epithelium (white arrow) seen on cytology smears from fine-needle aspiration (Diff-Quick stain).

The patient underwent an esophago-gastroduodenoscopy (EGD), which showed mild focal pre-pyloric edema and erythema. Biopsies were unremarkable. He continued to be symptomatic and an endoscopic ultrasound (EUS) was performed, which showed diffuse, moderately congested mucosa in the pre-pyloric region of the stomach, the pyloric channel and the proximal portion of the duodenal bulb. There was asymmetrical hypoechoic thickening of the wall of the pylorus, with loss of the normal five-layered echostructure in this region (Figure 3). Fine-needle aspiration (FNA) of the area of wall thickening was performed; five passes were made of the area with a 25 gauge needle. At no time did the needle penetrate the gastric serosal surface. Cytology showed bland pancreatic acinar cells with signs of chronic inflammation (Figure 4).

**Figure 5.**
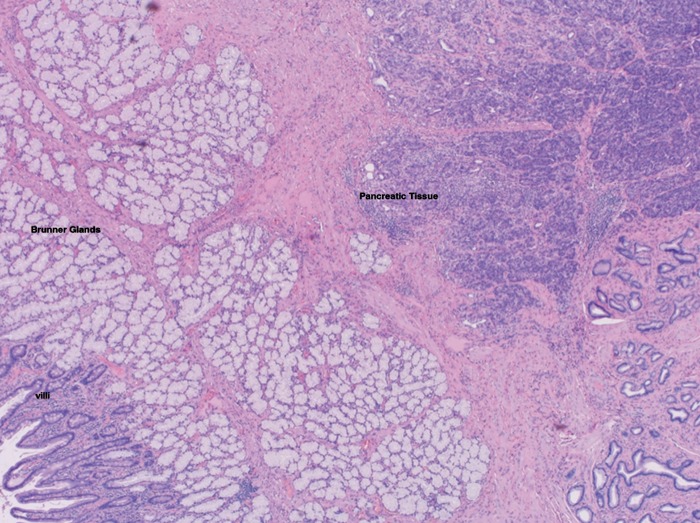
Representative section of surgical specimen, showing duodenal and ectopic pancreatic tissue (hematoxylin & eosin stain).

Inflammation within ectopic pancreatic tissue was suspected and, given his continued symptoms, weight loss and atypical appearance on CT and EUS, the patient was referred for surgical intervention. He underwent a distal gastrectomy. Given the degree of edema in adjacent duodenal tissue, a gastroduodenostomy was not feasible and, as a result, a Roux en y gastrojujenostomy was carried out. The pathology report was consistent with distal stomach with ectopic pancreas showing moderate chronic pancreatitis (Figure 5).

## Discussion

Heterotopic pancreas is a relatively common congenital anomaly that is reported in 1–2% of autopsy cases [1]. Ectopic pancreatic tissue most commonly occurs in the upper gastrointestinal tract, with occasional cases reported in the ilium, mediastinum, bile ducts, gall bladder, fallopian tubes, splenic hilum, omentum and lungs [3].

Heterotopic pancreas is usually asymptomatic and may be noted incidentally during endoscopy or imaging studies; it may become clinically evident when complicated by pathological changes such as inflammation, bleeding and malignant transformation. Symptoms would depend on the anatomical location of the ectopic tissue and the condition will manifest itself through obstructive symptoms or pain [2]. Lesions larger than 1.5 cm are thought to be clinically significant and associated with symptoms [4].

Histologically, ectopic pancreas is classified as Type 1, composed of acini, duct and islets, Type 2, composed of ducts only, Type 3 consisting of acini only (exocrine pancreas) and Type 4, which is composed of islets only (endocrine pancreas) [3].

Imaging studies, CT scans and EUS can all be utilized for evaluation of these lesions; however, pre-operative diagnosis can be difficult. On EGD, ectopic pancreas is noted as a firm, round, sub-epithelial lesion with a central depression that is thought to represent the opening of the duct. This last finding is present in less than 50% of cases, making it difficult to differentiate from other submucosal lesions, such as gastrointestinal stromal tumor (GIST) [3, 5]. The characteristic EUS features of ectopic pancreas are indistinct borders, lobulated margins, the presence of anechoic, duct-like structures, an intramural growth pattern, and localization within two or more layers [5]. Visualization of a pancreatic duct on magnetic resonance cholangiopancreatography may also provide clues to the diagnosis [6].

Despite the relatively common observation of ectopic pancreatic tissue during EGD, inflammation causing gastric outlet obstruction is exceedingly rare and limited to few case reports [1–3]. The management of these lesions would depend on their size, ability to exclude other etiologies and their associated symptoms. Given the inability to make a clear diagnosis, some patients require surgery. The etiology of acute or chronic pancreatitis of ectopic pancreatic tissue is seldom reported. In one report, alcohol was suspected to be the etiology of pancreatitis of ectopic pancreatic tissue without causing pancreatitis of the anatomical pancreas; however, this association could not proven [7]. Another case reported, in a patient with heavy alcohol use, the occurrence of pancreatitis simultaneously in ectopic and anatomical pancreatic tissues [8].

Our patient first presented with inflammation in an area of ectopic pancreas; he later developed an acute fluid collection in that area and continued to be symptomatic, requiring further evaluation and eventually surgery: pathology was consistent with Type 1 ectopic pancreas, with features of chronic inflammation. We speculate that heavy alcohol use may have caused his pancreatitis; however, this association cannot be proven. It is not clear at this time whether or not alcohol can cause ectopic pancreatitis without causing pancreatitis of anatomical pancreas. Although the most common cause of gastric outlet obstruction and elevated lipase without associated pancreatitis is penetrating peptic ulcer disease, other less-common etiologies should be considered.

*Conflict of interest statement*: none declared.
